# Why don't some women attend antenatal and postnatal care services?: a qualitative study of community members' perspectives in Garut, Sukabumi and Ciamis districts of West Java Province, Indonesia

**DOI:** 10.1186/1471-2393-10-61

**Published:** 2010-10-12

**Authors:** Christiana R Titaley, Cynthia L Hunter, Peter Heywood, Michael J Dibley

**Affiliations:** 1 Sydney School of Public Health, Edward Ford Building (A27), University of Sydney, Sydney, NSW 2006, Australia; 2 Menzies Centre for Health Policy, University of Sydney, Sydney, NSW 2006, Australia

## Abstract

**Background:**

Antenatal, delivery and postnatal care services are amongst the recommended interventions aimed at preventing maternal and newborn deaths worldwide. West Java is one of the provinces of Java Island in Indonesia with a high proportion of home deliveries, a low attendance of four antenatal services and a low postnatal care uptake. This paper aims to explore community members' perspectives on antenatal and postnatal care services, including reasons for using or not using these services, the services received during antenatal and postnatal care, and cultural practices during antenatal and postnatal periods in Garut, Sukabumi and Ciamis districts of West Java province.

**Methods:**

A qualitative study was conducted from March to July 2009 in six villages in three districts of West Java province. Twenty focus group discussions (FGDs) and 165 in-depth interviews were carried out involving a total of 295 respondents. The guidelines for FGDs and in-depth interviews included the topics of community experiences with antenatal and postnatal care services, reasons for not attending the services, and cultural practices during antenatal and postnatal periods.

**Results:**

Our study found that the main reason women attended antenatal and postnatal care services was to ensure the safe health of both mother and infant. Financial difficulty emerged as the major issue among women who did not fulfil the minimum requirements of four antenatal care services or two postnatal care services within the first month after delivery. This was related to the cost of health services, transportation costs, or both. In remote areas, the limited availability of health services was also a problem, especially if the village midwife frequently travelled out of the village. The distances from health facilities, in addition to poor road conditions were major concerns, particularly for those living in remote areas. Lack of community awareness about the importance of these services was also found, as some community members perceived health services to be necessary only if obstetric complications occurred. The services of traditional birth attendants for antenatal, delivery, and postnatal care were widely used, and their roles in maternal and child care were considered vital by some community members.

**Conclusions:**

It is important that public health strategies take into account the availability, affordability and accessibility of health services. Poverty alleviation strategies will help financially deprived communities to use antenatal and postnatal health services. This study also demonstrated the importance of health promotion programs for increasing community awareness about the necessity of antenatal and postnatal services.

## Background

Antenatal and postnatal care services are amongst the major interventions aimed at reducing maternal and newborn deaths worldwide [1-3]. Antenatal care services help pregnant women by identifying complications associated with the pregnancy or diseases that might adversely affect the pregnancy [2,4]. Through antenatal visits, women benefit from various interventions, including counselling about healthy lifestyles, the provision of iron/folic acid supplements, and tetanus toxoid vaccinations reported to protect newborns against neonatal death [1,2,4,5]. In Indonesia, pregnant women are recommended to receive at least four antenatal care checks, one in the first trimester, one in the second and two in the last trimester [6]. The minimum standard services provided include the measurement of body weight, blood pressure, symphysis-fundus height, Tetanus Toxoid (TT) vaccination, and iron/folic acid supplementation [6].

The postnatal period, just after delivery and through the first six weeks of life [4,7], is recognized as a critical time for both mothers and newborns. The importance of postnatal care services has been reported in various studies worldwide [8-10]. Postnatal care services enable health professionals to identify post-delivery problems, including potential complications, and to provide treatments promptly. In Indonesia, neonates are recommended to receive at least two adequate health care checks within the period of 0-7 days and 8-28 days after birth [11].

Different health service delivery modes, from facility-based clinical care to outreach and family and community care, will benefit mothers' and children's health [1]. In Indonesia, at the sub-district level, antenatal and postnatal care services are provided through the health centre, or *Puskesmas *(*pusat kesehatan masyarakat*), a primary health care level institution headed by a doctor or a public health officer [6,12]. *Puskesmas *is responsible for providing health services to the community within its service area. An inpatient care ward is available in some health centres, and is mostly used for delivery care services. Each *Puskesmas *usually has between three and five sub-health centres, called *Pustu (puskesmas pembantu)*. At the village level, the available health facilities include the *Pustu*, the integrated service post, called *Posyandu *(*pos pelayanan terpadu*), the village maternity post, called *Polindes *(*Pondok bersalin desa*), and village health posts, called *Poskesdes *(*Pos Kesehatan Desa*). *Posyandu *is a form of outreach service available at an administrative ward of a village and run voluntarily by the community (cadres). It provides maternal and child health services, including health counselling, physical examinations of pregnant women, nutrition, immunizations, as well as weighing of children under five years of age, all conducted on a monthly basis [12]. *Polindes *and *Poskesdes *are also forms of community-based activities for antenatal care services, including delivery and postnatal care conducted by village midwives [12]. As recommended by the WHO and UNICEF [13], the Indonesian Government has also promoted postnatal care in the form of home visitations conducted by trained birth attendants, although its implementation varies widely across the country [14].

There has been a series of attempts to improve the funding of health care, particularly for the poor. These efforts eventually resulted in the Health Insurance for the Poor scheme or *Asuransi Kesehatan Masyarakat Miskin (Askeskin) *in 2004, which evolved into the Community Health Insurance program or *Jaminan Kesehatan Masyarakat (Jamkesmas) *in 2008. These schemes aim to benefit disadvantaged citizens (identified from 14 criteria determined by Statistics Indonesia [15]) by providing free health care services, including antenatal, delivery, or postnatal care services [16,17]. Furthermore, in 2007 a conditional cash transfer program called *Program Keluarga Harapan (PKH) *was introduced and is currently being piloted in 40 districts throughout seven provinces, including some districts in West Java [18]. The PKH is aimed at increasing the education level and health status of the poor [18]. This means a cash allowance is provided to eligible recipients based on their compliance with certain conditions, such as the utilization of maternal and child health services.

Studies from developing countries [19-25] have reported the influence of demographic and socio-economic factors on the utilization of maternal and child health care services. Women with higher economic status [22,23], higher educational levels [19,22,24], and who live in urban areas [22] with adequate health care services [21,22,25] are more likely to utilize health care services. At the national level, previous analyses using various Indonesia Demographic and Health Survey (IDHS) data also confirmed the association of these factors with levels of antenatal or postnatal care service utilization [26,27].

Although the 2007 IDHS reported that 95% of pregnant women in Indonesia attended at least one antenatal visit, only 66% of mothers (58% in rural areas and 77% in urban areas) attended at least four antenatal care services as recommended. This figure was much lower than the national target of 90% antenatal care attendance [28]. Moreover, approximately 16% of mothers did not receive any postnatal care services (17% in the rural areas and 15% in the urban areas) [28]. The percentages of both antenatal and postnatal care uptake varied across provinces [6,28,29]. In South Sumatera province, the rates for antenatal care and postnatal care attendance were 70% and 43%, respectively; whereas in DI Yogyakarta the attendance rates were 97% and 82%, respectively [29].

The 2007 IDHS reported that in West Java province only 84% of mothers attended at least four antenatal services (tabulation was performed using the 2007 IDHS dataset [30]) and only 65% of mothers attended a postnatal service within two days of delivery [28]. Any evaluation of community perceptions about antenatal and postnatal care services, as well as the constraints of accessing those services, has the potential to improve maternal and neonatal health. This paper presents an analysis of community members' perspectives on antenatal and postnatal care services, including reasons for using or not using these services, and the health services received during antenatal and postnatal care in West Java province. Cultural practices (based on shared concepts, values, and ideals of a group) during antenatal and postnatal period were also explored. An analysis of the use of delivery care services in these study areas is presented elsewhere [31].

## Methods

### Sampling and study sites

The analysis presented here is part of a larger study aimed at exploring community members' perspectives on antenatal, delivery and postnatal care services. Community members include mothers and fathers of children aged one to four months, community health workers (cadres), traditional birth attendants, community and religious leaders, as well as health care providers such as village midwives. As mentioned earlier, the present analysis will focus on antenatal and postnatal services, as we have reported on delivery care services elsewhere [31]. Data used in this study were derived from the same set of focus group discussions and in-depth interviews.

This study was conducted in West Java province from March to July 2009. West Java is one of the most populous provinces of Indonesia with a total of 39 million people living in 17 districts and 9 municipalities [32]. The majority of the population are from the Sundanese ethnic group. Agriculture and industrial production are the main sources of livelihoods for the people in this area. Due to the proximity to the capital city, Jakarta, in some villages most of the women's husbands did not reside at home since they worked in the city and only returned home occasionally.

A purposive sampling method was used to select three districts, Garut, Sukabumi and Ciamis, representing a low, moderate and high rate of postnatal care service uptake, respectively [6,11]. The rate of postnatal care uptake in the first week after delivery was 25% in Garut, 51% in Sukabumi and 80% in Ciamis [32]. The rate of any antenatal care uptake was 75% in Garut; 94% in Sukabumi and 96% in Ciamis district. With assistance from the District Health Office staff, two villages were selected to represent areas with different levels of access to health services. A total of six villages were included in our study: Sukarame and Sukajaya villages (Garut district), Batu Nunggal and Limus Nunggal villages (Sukabumi district) and Benteng and Panyutran villages (Ciamis district).

### Participants

**Figure 1:**
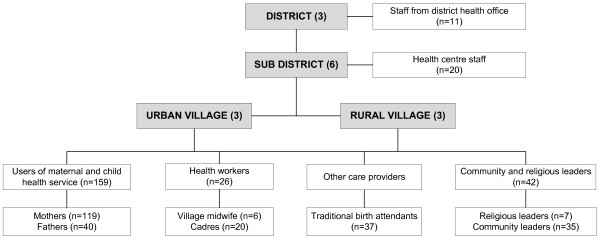
**Sampling frame for the qualitative study in West Java, Indonesia**.

To explore community members' perspectives on antenatal and postnatal care services, purposive sampling was employed to recruit 295 respondents, which consisted of 119 mothers and 40 fathers of children aged between 40 days and four months; 26 health care providers (i.e. nurses, midwives, village midwives); 20 local community health workers (cadres); 37 traditional birth attendants; 42 community and religious leaders; and 11 health office staff. Informed consent was obtained from each participant. The detailed sampling frame and respondents' categories are presented in Figure 1.

### Data collection

Focus group discussions (FGDs) and in-depth interviews were used to collect data from respondents. All interviews and FGDs were audio-recorded. During the activities, the interviewer or FGD facilitator was accompanied by a field assistant who also played a role as an observer. Six trained interviewers/facilitators and five field assistants were recruited and trained prior to data collection activities. The guidelines for discussions about antenatal and postnatal care services are presented in Table 1.

**Table 1:** Main topics included in the guidelines used for focus group discussions and in-depth interviews

	Topic	
	
Participant	During pregnancy	During postnatal period
**Mothers or fathers attending ANC^1 ^or PNC^2 ^services**	Type of ANC services received	Type of PNC services received
	Reasons for attending ANC services	Reasons for attending PNC services
	Frequency of ANC visits	Frequency of PNC visits
	Constraints of accessing ANC services	Constraints of accessing PNC services
	Satisfaction for using ANC services	Satisfaction for using PNC services
	Fee for ANC services	Fee for PNC services

**Mothers or fathers not attending ANC or PNC services**	Reasons for not attending ANC services	Reasons for not attending PNC services
	Constraints of accessing ANC services	Constraints of accessing PNC services

**Mothers or husbands using TBA^3 ^services during pregnancy or postnatal period**	The type of services received	The type of services received
	Reasons for using TBA services	Reasons for using TBA services
	Frequency of services received	Frequency of services received
	Satisfaction for using TBA services	Satisfaction for using TBA services
	Fee for the service	Fee for the service

**Health professionals**	Content of ANC services	Content of PNC services
	Frequency of ANC services	Frequency of PNC services
	Fee for ANC services	Fee for PNC services
	The implementation of ANC and PNC services in the community	
	Community attitude towards health professionals, ANC and PNC programs	

**Traditional birth attendants**	Type of services provided	Type of services provided
	Frequency of services	Frequency of services
	Fee for services	Fee for services

**Community and religious leaders**	MCH^4 ^programs in the community	
	Community attitude towards MCH programs and health professionals	
	Efforts in to increase MCH in the community	

**All participants**	Family support and decision making on health services during pregnancy and postnatal period	
	Traditional practices and beliefs during pregnancy and postnatal period	
	Perceptions about TBA, health professionals and health services provided to the community	

Note:

^1^ANC: antenatal care; ^2^PNC: postnatal care; ^3^TBA: traditional birth attendant; ^4^MCH: maternal and child health

On average, two FGDs were carried out in each village, one for women who attended at least two postnatal care services and/or were delivered by trained attendants; and another for women who did not attend at least two postnatal care services and/or were delivered by traditional birth attendants. Additional FGDs were conducted for traditional birth attendants in the mainly rural-type villages that rely on their services. In general, one FGD consisted of around seven participants, in addition to one FGD facilitator and one observer/assistant. FGDs were carried out either at a community hall or the house of one of the respondents.

In-depth interviews were also carried out with participants from different categories, as shown in Figure 1. Interviews were conducted within a private setting, often at the interviewee's house, to ensure the confidentiality of the responses and the convenience of the respondents, particularly those who had never had any contact with the health system.

Each participant received a cash payment of IDR 50,000 (~ USD 5.00) to cover their out of pocket expenditure for participating in the study. An information leaflet on maternal and child health care was provided to mothers, fathers, and traditional birth attendants at the end of each activity. The interviewer/facilitator and their assistant reviewed the process of interviews and FGDs regularly, and adjustments were made if necessary.

### Definitions

Antenatal care is defined as health care services related to pregnancy provided by skilled health personnel before delivery. This includes the provision of therapeutic interventions that would benefit the woman and her infant, as well as education about the importance of planning for a safe birth [33].

Postnatal care is defined as health services provided to mothers and newborns within the first 42 days after childbirth [34]. It includes early detection and treatment of complications and diseases as well as education about breastfeeding, immunization and nutrition [34].

### Data analysis

**Figure 2:**
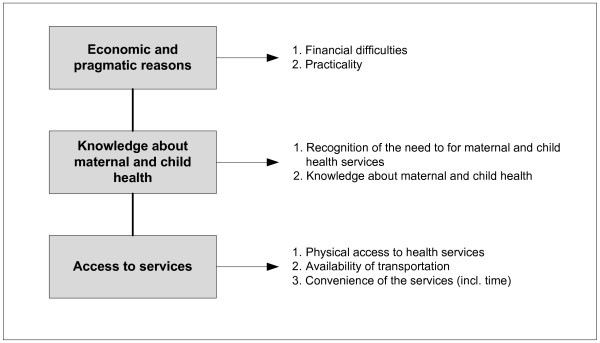
**Framework of factors influencing decision to use health services**.

We adapted the guideline developed by Thaddeus and Maine of factors influencing the utilization of health services [21]. Three main factors we identified were economic reasons, knowledge about maternal and child health, and access to services (Figure 2).

A de-identification process was conducted during data analysis to ensure the anonymity of respondents. Transcriptions of the interviews and FGDs were exported to NVivo 8 qualitative data analysis software, followed by a content and thematic analysis [35-37]. Data sources and triangulation methods [35-37] were performed to compare the responses from the community (mothers and fathers), care providers, and community leaders; as well as responses from in-depth interviews and FGDs.

### Ethical clearance

Ethical clearance for this study was obtained from the Human Research Ethics Committee (HREC) at the University of Sydney, Australia, and from the Ethical Research Commission National Institute of Health Research & Development, Ministry of Health Republic of Indonesia.

## Results

Issues regarding antenatal and postnatal care services are classified into five major topics, which are (1) Reasons for attending antenatal and postnatal care services; (2) Reasons for not attending antenatal or postnatal care services; (3) The practice of antenatal and postnatal care services; (4) Traditional practices during pregnancy and postnatal period; and (5) Community perceptions about midwives and traditional birth attendants.

### Reasons for attending antenatal and postnatal care services

The main reason for attending antenatal and postnatal care services was to ensure the safe health of mothers and infants. Some participants also mentioned other reasons such as problems during pregnancy or to follow other family members' experiences.

*We feel safe by attending antenatal services. We may know problems related to pregnancies. If we had never had our pregnancy checked, we would not be able to know any *[problems]. **(A mother, 26 years, in-depth interview, Batu Nunggal, Sukabumi District)**

*I went to see the village midwife because I had some bleeding in the 4th month *[of the pregnancy].* I was afraid I might miscarry*. **(A mother, 26 years, focus group discussion, Limus Nunggal, Sukabumi District)**

*I went to the midwife because my mother also used her service*. **(A mother, 23 years, in-depth interview, Batu Nunggal, Sukabumi District)**

Another reason for attending postnatal care services mentioned by the participants was immunization for the newborns.

### Reasons for not attending antenatal or postnatal care services

#### Economic and pragmatic reasons

Our study found that the perceived cost of health services emerged as a major issue hindering community members from utilizing antenatal and postnatal care services.

*I went to traditional birth attendants. It is cheaper. I think you have to pay *[to have your pregnancy checked] *in Posyandu *[integrated service post].* I will go there if I do not need to pay anything*. **(A mother, 22 years, in-depth interview, Panyutran, Ciamis District)**

*They said they did not want to have their pregnancy checked because they did not have any money. Some said they needed more money to use a midwife's services. It is different from traditional birth attendants. You do not need much money to pay them*. **(A cadre, in-depth interview, Limus Nunggal, Sukabumi District)**

Additionally, transportation costs added to the burden. Some participants mentioned the reason of practicality for using the services of traditional birth attendants as they lived closer than health care providers.

*The problem is I did not have any money to pay the transport. I want to have my pregnancy checked by the doctor or the midwife every month, but their places are so far away. I needed transport to get there. Instead, I went and sought traditional birth attendants*. **(A mother, 22 years, in-depth interview, Sukarame, Garut District)**

Although *Jamkesmas *cards had been provided to some poor and near poor communities to enable them to use health services for free, misconceptions about its eligibility were found in the villages. Some participants stated that *Jamkesmas *cards could be used only for particular health care providers, such as the village midwife; or health care services, such as delivery services; some did not think they could be used for antenatal and postnatal services.

*Jamkesmas does not cover health care services after delivery; it is only for delivery services*. **(A mother, 29 years, in-depth interview, Sukarame, Garut District)**

*You can only use Jamkesmas with the village midwife and nothing other than that*. **(A cadre, in-depth interview, Batu Nunggal, Sukabumi District)**

*Jamkesmas... sometimes you can, but sometimes you cannot use it... They said Jamkesmas was useless... when you bring it to the doctor it *[the service] *is still expensive*. **(A father, focus group discussion, Panyutran, Ciamis District)**

Moreover, our study found that free health services were assumed to be associated with a lesser quality of both health services and medications compared to health services that required some payments.

*I do not have any money. For you to have your pregnancy checked, you need much money. It is free in the health centre, but it is better to go to the services where you can pay. They will give you better medication*. **(A mother, 34 years, focus group discussion, Sukarame, Garut District)**

#### Knowledge about maternal and child health

Some participants did not feel the need to have antenatal or postnatal care services as they did not experience any problems during pregnancy and after delivery.

*I did not go to the midwife anymore *[within 40 days after delivery]. *I felt healthy*. **(A mother, 20 years, focus group discussion, Limus Nunggal, Sukabumi District)**

*I feel healthy. Nothing happened. You need money to go the midwife. If I am not sick why should I go to the midwife *[after delivery]? *I do not want to waste my money*. **(A mother, 22 years, in-depth interview, Panyutran, Ciamis District)**

*They do not want to come usually because they feel healthy and there is no complication with the pregnancy. If they have some problems, usually they will come*. **(A health centre midwife, in-depth interview, Sukarame, Garut District)**

Furthermore, negative attitudes towards child immunization were mentioned as a reason for not attending postnatal care services.

*Someone told me to bring my child for immunization. But you know, after immunization children usually have some swelling on their hands. Sometimes it is like an abscess. The child will cry, and get sick. I do not want it. I am afraid*. **(A mother, 22 years, in-depth interview, Panyutran, Ciamis District)**

*I was afraid my child would have a fever if we brought him to the midwife. They said usually children will suffer from fever after immunization. I do not want him to get sick*. **(A cadre, in-depth interview, Sukarame, Garut District)**

#### Access to services

Physical proximity to health services was a major problem, especially in rural villages with poor road conditions. Some participants complained that they needed to walk for up to two hours to reach the nearest health centre. The situation became worse during the rainy season when the road was slippery.

*It is really hard when it is raining. We are afraid we will fall over because the road is so slippery and we are pregnant. The health centre is far and you can see that the road condition is so poor*. **(A mother, 36 years, focus group discussion, Limus Nunggal, Sukabumi District)**

Another constraint was the limited availability of health services, particularly in remote areas, where the village midwife either did not live there or frequently travelled out of the village.

*To be honest, there are new midwives who do not live in the village; some of them are also doing further study in midwifery *[in the city]. **(A health centre midwife, in-depth interview, Sukarame, Garut District)**

*The village midwife is currently studying in the capital city. So she is not available from Friday to Sunday*. **(A mother, 26 years, focus group discussion, Limus Nunggal, Sukabumi District)**

Most women in these villages are agricultural workers during daylight hours. This means attending health care services during the daytime, compounded by long waiting periods, might lead to loss of income.

### The practice of antenatal and postnatal care services

The Government's guidelines for antenatal and postnatal care services had been implemented in all villages. The minimum of four antenatal care visits and two postnatal visits were encouraged.

In these study areas, antenatal services were mainly provided at *Puskesmas *and monthly in *Posyandu*. Antenatal care services included physical examinations, weight measurements, counselling, Tetanus Toxoid vaccinations, as well as iron/folic acid supplements. Ninety tablets of iron/folic acid supplements are recommended for pregnant women. However, not all women in the villages reported receiving 90 tablets during pregnancy. Some participants mentioned that the supplements were received every time they visited antenatal care services. The number of supplements women received varied between 15 and 30 tables. Consequently, women attending antenatal care less than four times received a fewer number of supplements. The experience of adverse effects, such as nausea, and traditional beliefs about the effect of supplements became reasons for failing to comply with the national guidelines.

*I was afraid I would have a large baby. It *[the iron/folic acid supplement] *also has a bad smell; I could not stand it, so I did not take it. Sometimes I finished the whole package in a month, sometimes I did not*. **(A mother, 37 years, focus group discussion, Benteng, Ciamis District)**

Although postnatal care services in the form of home visitations have been endorsed, the practice varied widely across villages. Some participants reported that they were never visited by the midwife within the first month after delivery. Postnatal services were also provided by *Posyandu *and *Puskesmas*. According to study participants who attended postnatal care services, they received such services as BCG, Hepatitis B and Polio immunization for the baby, cord care, physical examination, as well as counselling about breastfeeding and infant health care.

*Three days after delivery, the village midwife visited and examined my body as well as the baby's. She examined the baby's navel, gave immunization, and vitamin A*. **(A mother, 30 years, focus group discussion, Batu Nunggal, Sukabumi District)**

Participants raised concerns about the availability of newborn immunization vaccines, particularly the BCG vaccine that was not always available, leading to a delayed immunization schedule. Community understanding about immunizations was still lacking, as some study participants did not know the type and benefit of immunization received by their infants.

### Traditional practices during pregnancy and postnatal period

The services of traditional birth attendants were commonly used in all villages during antenatal and postnatal periods. Traditional birth attendants massaged mothers, usually in the fourth and eighth month of pregnancy, using traditional herbal medicine such as coconut oil, and holy water.

*The traditional birth attendant massaged me when I was four, seven and eight or nine months pregnant... you know sometimes it was a breech position. They know the baby's position*. **(A mother, 25 years, in-depth interview, Batu Nunggal, Sukabumi District)**

*Usually in the fourth month of pregnancy, there will be a traditional ritual held. We will be called to massage the pregnant mother using coconut oil*. **(A traditional birth attendant, focus group discussion, Sukajaya, Garut District)**

After delivery, traditional birth attendants provided a regular service to newly-delivered mothers and newborns. Daily visits were conducted to bath the newborn and to treat the newborns' umbilical cord until it fell off. Mothers would be massaged to hasten the return of the uterus to its normal size. Additional visits occurred to check both the mother's and infant's condition until the 40^th ^day after delivery.

*I come every day until the umbilical cord falls off. I put Betadine and usually after three days it falls off. Before that I only wipe the newborn... I will give him/her a bath after the umbilical cord falls off*. **(A traditional birth attendant, in-depth interview, Sukarame, Garut District)**

### Community perceptions about midwives and traditional birth attendants

In remote areas where one village midwife was available, traditional birth attendants were more capable of reaching the community. Their role was also perceived to be important by the community.

*Some people said traditional birth attendants are more patient and careful. They could visit the mother and infant until 40 days after delivery. For seven days after delivery, the traditional birth attendants will come to bath the baby until the umbilical cord falls off. The mother will be massaged as well. And some said that the traditional birth attendants were still preferable, because they were cheaper*. **(Staff of district health office, in-depth interview, Ciamis District)**

However, we also found some women preferred using the service of health professionals, such as the village midwife, over the traditional birth attendants due to better equipment or more thorough examinations.

*Traditional birth attendants had an incomplete set of equipment, while for the village midwife they already have a complete one. To be safe*. **(A mother, 36 years, focus group discussion, Limus Nunggal, Sukabumi District)**

*I sought the midwife's service because she checked us more carefully, not like the traditional birth attendants who only touched us*. **(A mother, 28 years, focus group discussion, Limus Nunggal, Sukabumi District)**

From the traditional birth attendants, we found a positive response about working together with the village midwife.

*I told them *[the women] *if you want to stay healthy, you need to be examined by the midwife. You need to be treated and examined during the Posyandu service*. **(A traditional birth attendant, in-depth interview, Sukajaya, Garut District)**

The roles of both traditional birth attendants and village midwives were also considered to be important in the community.

*We need both the traditional birth attendant and the midwife. The traditional birth attendant can massage, fix the baby's position and the stomach muscles of a pregnant woman...I haven't encountered a midwife that could do that. Midwives usually give medications. So, both of them are still needed*. **(A community leader, in-depth interview, Panyutran, Ciamis District)**

## Discussion

### Main findings

Positive attitudes about the antenatal and postnatal care services were found in all villages. The reasons for attending these services were mainly to ensure the safe health of both mothers and infants. Financial difficulty was a major issue for women who did not attend any antenatal or postnatal care services as recommended. Although some poor and near poor communities received *Jamkesmas *cards, misconceptions about its use and the insurance scheme emerged. Physical distance to health facilities aggravated by poor road conditions (especially during rainy season) hindered women and newborns from receiving antenatal and postnatal care services. In remote areas, the limited availability of health services is a constraint for service uptake, especially where the village midwife frequently travels out of the village or does not live there. Furthermore, we found a lack of awareness about the importance of maternal and child health care services. Participants only perceived health care services to be necessary if obstetric complications occurred. In general, the services of antenatal and postnatal care were provided as recommended. Home visitation for postnatal care services was effective in providing health care for those who had never been in any contact with the health system. Our study found that traditional birth attendants played a strategic role either during pregnancy or post-delivery. Their services were considered essential and were highly utilized in some communities.

### The use of antenatal and postnatal services

Some women in the villages did not use any antenatal care services or postnatal care services even though the services were available at the village level, as reported by other studies from West Java [38,39]. Financial difficulties limit the community's ability to use these services. This finding is confirmed by previous studies from developing countries, which demonstrate that communities with low household wealth were more likely not to use health care services [22,27,40,41]. The provision of *Jamkesmas *cards does not automatically improve the community's health seeking behaviour. Some still do not attend antenatal or postnatal care services, or both. Limited access to information, especially among those who had less frequent contact with health providers or other village authorities, might be linked to a lack of understanding about the use of the *Jamkesmas*. This finding is supported by an earlier study conducted in Banten Province, Indonesia, [42] which revealed the community's lack of knowledge of the insurance scheme for the poor. This was found not only among the families, but also midwives [42]. These research findings indicate the importance of conducting appropriate promotional programs to improve community knowledge and understanding about the benefits of *Jamkesmas*. The conditional cash transfer scheme or *PKH*, which is still being piloted in Indonesia, might be an alternative strategy to increase the uptake of maternal and child health services, as shown in Mexico and Honduras [43,44]. This could be more effective than the unconditional cash transfer program, or *Bantuan Langsung Tunai *[15]. These healthcare-financing strategies, however, should be accompanied by regular evaluation and a monitoring system to assess their effectiveness in reaching target populations and changing their health behaviours.

Previous literature has reported that utilization of health services is strongly associated with access to health services [21,22,45-47]. Despite efforts to bring health services closer to the community, physical distance remains a major problem in these areas. The remoteness from health facilities increased community members' out-of-pocket expenditure for transportation costs. The opportunity costs lost due to travel and waiting time were constraints to the uptake of services. The recommended home visitation for postnatal care services [13] will greatly benefit mothers and newborns, especially those living in isolated areas. Moreover, the outreach health service for antenatal care could also be an alternative in these settings.

The limited availability of village midwife services in rural areas was due, in part, to the limited coverage of their services, especially in sparsely populated areas. It was also a result of midwife absenteeism, as they frequently travel out of the village or do not reside there [14]. A study from Banten province showed that less than 30% of village midwives resided in their assigned villages [48]. With the growth of the private sector, a village midwife who receives a government salary also wants to work as a private practitioner [39]. This means that living in urban areas is preferable and profitable for private practice. A significant association between distance to urban areas and the increased income for midwives was confirmed in another study [49]. Further, some experienced midwives prefer to live in a nearby town to avoid professional isolation and to maximize access to career development opportunities [14,48].

Different strategies have been implemented to keep village midwives in remote areas, including income supplements or a renewal rolling contract [48]. However, retaining midwives in the village remains an enormous challenge, and failure to do so will lead to a lower coverage of current health services than has currently been achieved, particularly as the village midwife is usually a solo health care provider. The use of a team of providers, such as midwife and midwife assistant, might be considered in order to increase the coverage of their services [50].

A study using a special part of the IDHS 2002/3 and 2007 demonstrated that the majority of women received antenatal care from private sector providers, such as a nurse, a midwife or a village midwife [51]. This increased usage of the private sector implies potential problems for the poor and those living in rural and remote areas. Moreover, a recent review of maternity skilled care showed that the increase of maternal care services provided by health professionals was mainly found in urban areas, whereas rural areas experienced a stagnation of professionalization of childbirth [50]. An Indonesian study also demonstrated a lower midwife density in rural than in urban areas [48]. Furthermore, village midwives who were assigned to rural and remote areas were those with less experience [48]. All of these findings require urgent attention from the central and local government sector to ensure social and economic equity of health services.

Our findings that demonstrate a lack of understanding among community members concerning the importance of maternal and child health care services are echoed in another Indonesian study [52]. Since pregnancy and childbirth were considered a woman's natural rite of passage, some might think that seeking medical attention is only for those experiencing obstetric complications [25]. This confirms the need to develop health promotion programs to raise community awareness about the protective role of these services. Some respondents demonstrated a lack of knowledge or misconceptions about the importance of antenatal and postnatal care service components, such as the use of iron/folic acid supplements or the type and benefit of immunizations. This indicates a need to strengthen programs for health education. Programs that target not only women but also other family members, such as husbands and parents, might increase awareness about the role of maternal and child health services, as shown in other literature [53-55].

### The role of traditional birth attendants

The services of traditional birth attendants for maternal and child care have been recognized for a long time prior to the introduction of the village midwife program in Indonesia. Even today, in some communities, traditional birth attendants' services are highly utilized due to trust and every day cultural practices in the community. This is also due to better access, particularly in remote areas where traditional birth attendants outnumber the village midwife. There is a strong attachment to these attendants and their services; they were also preferred in the event of an emergency during the postnatal period [48]. Studies have shown that the involvement of traditional birth attendants in the health care system has improved maternal and perinatal health [56,57]. Traditional birth attendants could, therefore, be empowered through training activities to provide safe health care services to mothers and infants, under the supervision of health professionals. Moreover, the partnership program between midwife and traditional birth attendants, which is currently focused on delivery care services [58], might be expanded to include antenatal and postnatal care services. The collaboration between these providers in the village might benefit women in areas where traditional birth attendants have a prominent role.

### Community involvement in promoting the use of antenatal and postnatal care services

Our study shows that public health strategies to promote the use of antenatal and postnatal care services are required in these communities. Efforts to strengthen community-based participatory programs might help to improve health service uptake, as shown in other studies [59-61]. Local community members could be encouraged to become actively involved. One example is the *SIAGA *(alert) program, an initiative that engages local community members to participate in maternal and child health programs [62]. This program helps women from households with low economic status to access maternal and child health services through a communal financing scheme, or by organizing transportation to more fully equipped health facilities [62]. The benefits of involving local community members through initiatives like the *SIAGA *program have been reported elsewhere [31].

### Strengths and limitations

Our study provides data about stakeholders' perspectives of antenatal and postnatal care services at the community level. This could inform policy makers to develop strategies to increase service uptake. The results are not intended to be representative of all provinces on Java Island. In this qualitative study, a purposive sampling method was employed with a small sample size that provides depth, rather than breadth, of understanding. The use of multiple interviewers, different data collection techniques and different categories of respondents increases the validity of the study [35].

This study has a number of limitations. It did not explore the quality of antenatal and postnatal care services delivered to the community, such as the type of information and health education provided to women. Further investigation is, therefore, needed to examine these issues. Language barriers might also be a disadvantage during data collection, although all research assistants played a role as an interpreter for the interviewer or respondents. Nevertheless, the validity of the study's results is unlikely to be affected by those issues.

## Conclusions

Antenatal and postnatal services were still under-utilized, despite community members' positive attitudes regarding these services. The factors that hindered utilization of antenatal and postnatal care services in our study included financial difficulties, physical distance to health facilities aggravated by poor road infrastructure, a limited availability of health services, and perceived need for health services. Misunderstanding about the eligibility of *Jamkesmas *has also prevented poor communities from fully benefitting from this insurance scheme. For some women who perceived pregnancy and delivery as a natural process in life's events, the services of the traditional birth attendants were part of their cultural practices. Unless obstetrics complications arose, there was no perceived necessity in using health professional services.

No 'magic bullet' solution is available to overcome the constraints; instead, comprehensive public health approaches are required. Poverty alleviation strategies will help financially deprived communities to access and use maternal and child health services. Appropriate socialization programs about *Jamkesmas *are important to ensure its optimum utilization among poor and near poor communities. In addition, evaluation and monitoring programs about its benefit and effectiveness should be conducted regularly.

Strategies that address problems related to the limited availability of health services should be a priority. This includes efforts to retain village midwives in isolated areas, as well as the use of a team of providers, such as a midwife and midwife assistants, to increase the coverage of their services. The involvement of traditional birth attendants might be an alternative solution for providing basic antenatal and postnatal services under the supervision of health professionals.

Health programs aimed at increasing community awareness about the importance of antenatal and postnatal services should be considered. Strengthening community-based participatory programs to actively engage in overcoming constraints will be beneficial. Local community members should also be involved to encourage pregnant women and newly delivered mothers to use health services.

## Competing interests

The authors declare that they have no competing interests.

## Authors' contributions

All authors designed the study. CRT conducted data collection. Under the supervision of CLH, CRT conducted data analysis and wrote the first draft of the manuscript. MJD and PH provided data analysis advice and revision of the final manuscript. All authors read, commented on and approved the final manuscript.

## Pre-publication history

The pre-publication history for this paper can be accessed here:

http://www.biomedcentral.com/1471-2393/10/61/prepub
